# Clinical characteristics and treatment outcomes of primary malignant melanoma of esophagus: a single center experience

**DOI:** 10.1186/s12876-022-02235-8

**Published:** 2022-03-29

**Authors:** Tae-Se Kim, Byung-Hoon Min, Yang Won Min, Hyuk Lee, Poong-Lyul Rhee, Jae J. Kim, Jun Haeng Lee

**Affiliations:** Department of Medicine, Samsung Medical Center, Sungkyunkwan University School of Medicine, 81 Irwon-ro, Gangnam-gu, Seoul, 06351 Korea

**Keywords:** Disease attributes, Melanoma, Treatment outcome, Survival

## Abstract

**Background:**

Primary malignant melanoma of esophagus (PMME) is an extremely rare disease with poor prognosis. We aimed to determine the clinical characteristics and treatment outcomes of patients with PMME.

**Methods:**

We retrospectively reviewed 17 patients diagnosed with PMME in Samsung Medical Center between 2000 and 2020 with median 34 months of follow-up. Survival outcomes were analyzed with Kaplan–Meier method.

**Results:**

15 patients (88.2%) were male and the most common presenting symptom was dysphagia (9/17, 52.9%). On endoscopy, tumors were mass-forming in 15 patients (88.2%) and diffusely infiltrative in two patients (11.8%). Lesions were melanotic in 13 patients (76.5%) and amelanotic in four patients (23.5%). The most common tumor location was lower esophagus (11/17, 64.7%). The disease was metastatic at the time of diagnosis in four patients (23.5%). As for treatment, 10 patients (58.8%) underwent surgery. In all 17 patients, the median overall survival was 10 months. In surgically treated patients, all patients experienced recurrence and the median disease-free survival was 4 months. There was no statistical difference in overall survival between patients with or without surgery. Patients with diffusely infiltrative tumor morphology had better overall survival compared to those with mass-forming tumor morphology (*P* = 0.048). Two patients who received immunotherapy as the first-line treatment without surgery showed overall survival of 34 and 18 months, respectively.

**Conclusions:**

As radical resection for patients with PMME does not guarantee favorable treatment outcomes, novel treatment strategy is required. Further large-scale studies are warranted to determine the efficacy of immunotherapy for patients with PMME.

## Background

Primary malignant melanoma of esophagus (PMME) is an exceedingly rare disease entity which is estimated to comprise only 0.1–0.2% of all esophageal malignancies [1]. PMME is known to behave aggressively and the estimated median overall survival is reported to be 10–12 months [2, 3], irrespective of treatment modalities. The mainstay of treatment for PMME has been esophagectomy because previous studies with limited number of cases suggested extended survival after radical resection [4–7]. However, due to the extreme rarity of the disease, its clinical features are sparsely reported and treatment strategies are not standardized.

Recently, several studies reported the efficacy of immunotherapy in patients with mucosal melanoma. In CheckMate 067 study including 79 advanced mucosal melanoma patients treated with immune checkpoint inhibitor, the five-year overall survival rate was 36% and the median overall survival was 22.7 months in the combination immunotherapy group (Nivolumab plus Ipilimumab), which was better than those of either monotherapy group [8]. For PMME, there have been only a few small-sized studies on the outcomes of immunotherapy [9–11]. In the largest study by Wang et al. [11] (n = 12) patients who received programmed death (PD)-1 inhibitors for PMME showed mean progression-free survival of 15.6 months. More studies with consistent results are required to validate the efficacy of immunotherapy for PMME.

In the present study, we reviewed the clinical and endoscopic features of 17 patients diagnosed with PMME in our institution and investigated their surgical and non-surgical outcomes.

## Methods

### Research design and study population

We retrospectively reviewed patients who were diagnosed with PMME between January 2000 and December 2020 at Samsung Medical Center. Only the patients with histologic confirmation of malignant melanoma in either biopsy or surgical specimen of esophagus were included. Patients with concurrent or a history of melanoma in other sites (including skin) were excluded. The study protocol was approved by the Institutional Review Board (IRB) of Samsung Medical Center (approval number: 2021–09-030–001) and conducted in accordance with the guidelines of the Declaration of Helsinki. Because of the retrospective nature of the study, written patient consent was waived by the IRB.

### Variables, data sources, and measurements

Clinicopathological data were extracted from the intranet database of Samsung Medical Center. Two board-certified gastroenterologists (T.S.K. and B.H.M.) thoroughly reviewed the medical records and endoscopic findings. The gross findings were categorized into two patterns: mass-forming and diffusely infiltrative. Anatomical location was defined as upper (20–25 cm from the incisor teeth (IT)), middle (IT 25–30 cm), and lower (IT > 30 cm) esophagus [12]. Because there is no standardized method of tumor staging for PMME, we categorized the patients into three staging categories with regard to lymph node metastases (LNM) and distant metastases status: localized disease (no LNM, N0), node positive disease (positive LNM, N+), and metastatic disease (M1) (adopted from Weiner et al. [3]). Surgical techniques were the same as those for patients with esophageal squamous cell carcinoma. Detailed description of surgical techniques used in our institution is reported elsewhere [13]. The survival time was calculated from the date of PMME diagnosis to the date of death or to the last date of follow-up (cut-off date: July 31, 2021). In patients who were lost to follow-up, survival data were retrieved from the National Health Insurance System Database. The disease-free survival time for patients who underwent surgery was calculated from the date of surgery for PMME to the date of first recurrence noticed during routine surveillance by computed tomography or esophagogastroduodenoscopy. Chemotherapy responses were measured according to the Response Evaluation Criteria for Solid Tumors (RECIST) version 1.1 [14]. In six patients, polymerase chain reaction (PCR) sequencing for BRAF mutation (exon 15) was performed. In three patients, immunohistochemical (IHC) staining for programmed death-ligand 1 (PD-L1) was performed, which was expressed as tumor proportion score (TPS): the percentage of viable tumor cells showing partial or complete membrane staining for PD-L1.

### Statistical analysis

Baseline clinicopathologic characteristics were summarized in mean ± standard deviation or frequency (percent). The Kaplan–Meier survival curve was plotted for the whole study population and the differences between patient groups were tested using a log-rank test. The median follow-up time was calculated using the reverse Kaplan–Meier method. Statistical significance was set at *P* < 0.05. All analyses were performed using SPSS version 25.0 (IBM SPSS Statistics for Windows, Version 25.0. Armonk, NY: IBM Corp.)

## Results

### Clinical characteristics of the patients

**Fig. 1:**
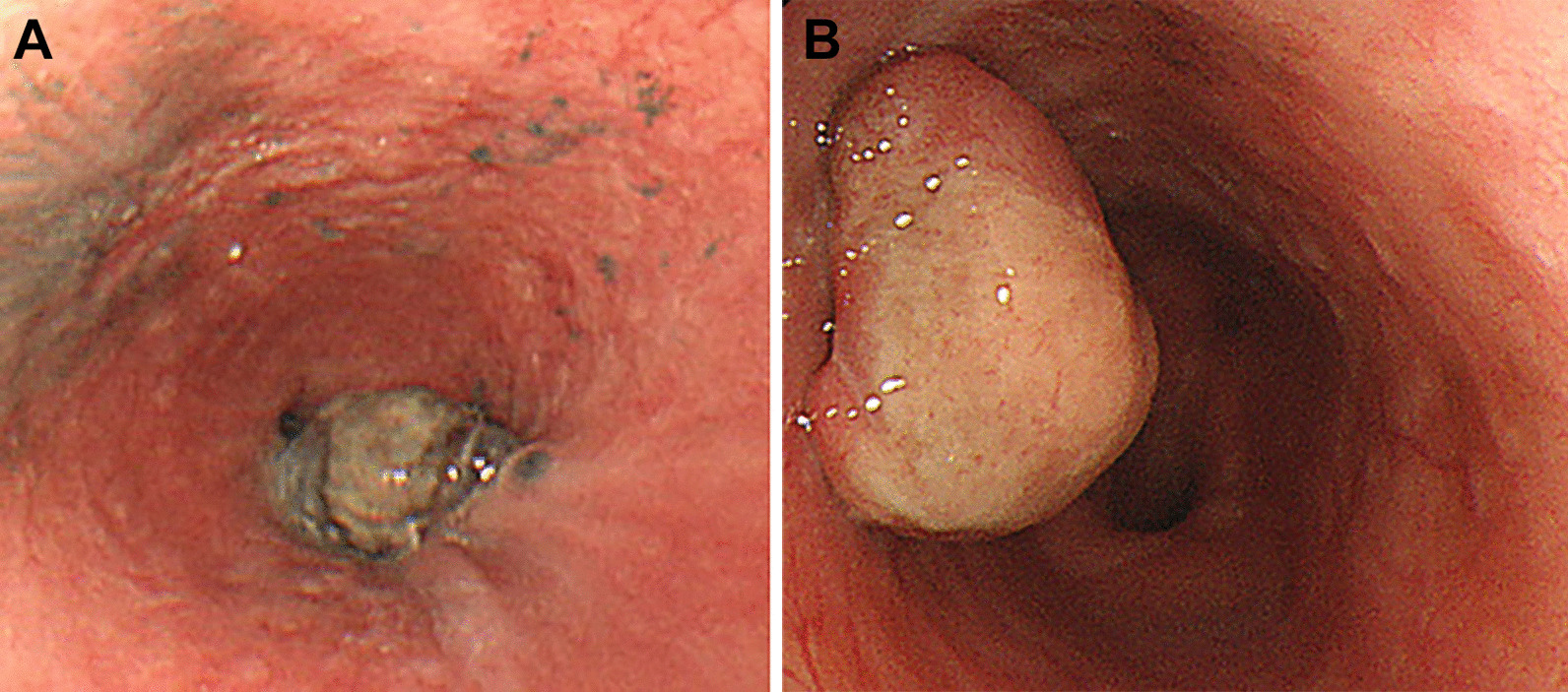
Representative images of melanotic and amelanotic type of primary malignant melanoma of esophagus. It typically presents as dark pigmented mass at lower esophagus (**A**). However, the absence of dark pigmentation in endoscopic examination does not exclude the possibility of malignant melanoma of esophagus (**B**)

The clinical characteristics of 17 patients with PMME are summarized in Table 1. The median age was 60 years (range: 41–83) and 88.2% (15/17) were male. The most common presenting symptom was dysphagia (52.9%, 9/17). Endoscopically, 15 cases (88.2%) presented with a mass-forming lesion, and 2 cases (11.8%) were diffusely infiltrative. 13 cases (76.5%) had dark pigmentation on endoscopic examination (Fig. 1A) and four cases (23.5%) did not (Fig. 1B). In the majority (64.7%, 11/17) of cases, tumors were located at the lower esophagus. Clinical staging was as follows: localized disease (8/17, 47.1%), node positive disease (5/17, 29.4%), and metastatic disease (4/17, 23.5%).

**Table 1:** Baseline characteristics of patients with primary malignant melanoma of esophagus

	Total (n = 17)
Age (years)	
Mean ± SD	61.0 ± 12.3
Median (range)	60 (41–83)
Sex (%)	
Male	15 (88.2)
Female	2 (11.8)
Chief complaint (%)	
Dysphagia	9 (52.9)
Epigastric discomfort	2 (11.8)
Chest pain	1 (5.9)
Dyspepsia	1 (5.9)
Nausea	1 (5.9)
Weight loss	1 (5.9)
No symptom	2 (11.8)
Smoking (%)	
No	9 (52.9)
Yes	8 (47.1)
Alcohol ingestion (%)	
No	6 (35.3%)
Yes	11 (64.7%)
BMI (kg/m^2^)	
Mean ± SD	23.8 ± 2.6
Median (range)	23.7 (19–30)
Endoscopic morphology (%)	
Mass-forming	15 (88.2)
Diffusely infiltrative	2 (11.8)
Amelanotic type (%)	
No	13 (76.5)
Yes	4 (23.5)
Endoscopic size* (cm)	
Mean ± SD	4.8 ± 2.2
Median (range)	5.0 (2–10)
Endoscopic location (%)	
Upper	3 (17.6)
Middle	3 (17.6)
Lower	11 (64.7)
Clinical staging (%)	
Localized (N0)	8 (47.1)
Node positive (N+)	5 (29.4)
Metastatic (M1)	4 (23.5)
Surgery (%)	
No	5 (29.4)
Yes	10 (58.8)
Follow-up loss	2 (11.8)

*SD* standard deviation, *BMI* body mass index

*Two patients with diffusely infiltrative type tumor were excluded

### Treatments and outcomes

Among the 17 patients, 10 (58.8%) received surgery, five (29.4%) received chemotherapy or palliative care, and two (11.8%) were lost to follow-up without treatment.

The outcomes of 10 patients who underwent surgical treatment are summarized in Table 2. The majority (70%) of patients received Ivor-Lewis operation. In the surgical specimen, the mean tumor size was 5.4 ± 2.8 cm. The invasion depth was limited to submucosal layer in seven cases (70%) while the muscularis propria was invaded in three cases (30%). Resection margin was negative in all patients and LNM was identified in six patients (60%). Post-operative complications were noticed in two patients (20%). One patient had post-operative chylothorax and was successfully treated with thoracic duct ligation surgery (number 1 in Table 2). The other patient had transient vocal cord hypomobility which gradually improved in 3 months with rehabilitative training (number 9 in Table 2). Two patients who survived longer than three years (patient number 1 and 2 in Table 2) did not have LNM. With regard to adjuvant therapy, three patients received intravenous interferon-alpha (IFN-α) and two patients received adjuvant Pembrolizumab (TPS of PD-L1 was 30% and 1% for patient number 3 and 6 in Table 2, respectively). Patients who received adjuvant IFN-α or adjuvant Pembrolizumab remained disease-free for 4, 4, and 1 months and 4 and 3 months, respectively. Apart from one patient lost during follow-up (patient number four in Table 2), recurrence was noticed in all patients who received surgery. Anastomosis (33%) and peritoneum (33%) were the most common sites of recurrence. The Kaplan–Meier estimate of median recurrence-free survival of surgically treated patients was only 4 months.

**Table 2:** Surgical outcomes for patients with primary malignant melanoma of esophagus

No	Age	Sex	Surgery type	Tumor location	Tumor morphology	Tumor size (pathology, cm)	Tumor depth (pathology)	LNM	Resection margin	BRAF	PD-L1 (TPS)	Adjuvant therapy	Disease-free survival (months)	Recurrent organ	Overall survival (months)	Outcome
1	48	M	3-field	Upper	DI	0.8	SM	0/13	Negative	Not detected	N/A	No	36	Anastomosis	59	Dead
2	53	F	I-L	Middle	MF	7.5	SM	0/33	Negative	Not detected	N/A	No	15	Femur, lung	38	Dead
3	70	M	I-L	Lower	MF	8.5	PM	2/46	Negative	N/A	30%	Pembrolizumab	4	Peritoneum	25	Alive
4	41	F	I-L	Lower	MF	2.2	SM	3/21	Negative	N/A	N/A	N/A	N/A	N/A	22	Alive
5	65	M	TG	EGJ	MF	4.0	SM	9/40	Negative	N/A	N/A	IFN-α	4	Anastomosis, liver	11	Dead
6	51	M	I-L	Lower	MF	6.0	PM	4/17	Negative	Not detected	1%	Pembrolizumab	3	LN, peritoneum, abdominal wall	9	Dead
7	77	M	I-L	Lower	MF	9.0	PM	9/25	Negative	N/A	N/A	No	5	Anastomosis, liver, peritoneum	8	Dead
8	69	M	I-L	Lower	MF	3.5	SM	0/7	Negative	Not detected	N/A	IFN-α	4	Neo-esophagus	7	Dead
9	57	M	3-field	Middle	MF	4.5	SM	5/80	Negative	Not detected	N/A	RT followed by IFN-α	1	Supraclavicular LN	6	Dead
10	53	M	I-L	Lower	MF	3.5	SM	0/36	Negative	N/A	N/A	No	4	Brain	6	Dead

*LNM* lymph node metastases, *PD-L1* programmed death-ligand 1, *TPS* tumor proportion score, *M* male, *F* female, *DI* diffusely infiltrative, *MF* mass-forming, *I-L* Ivor-Lewis operation, *TG* total gastrectomy, *EGJ* esophagogastric junction, *SM* submucosa, *PM* muscularis propria, *RT* radiotherapy, *IFN-α* interferon-alpha, *N/A* not available

The outcomes of five patients who did not undergo surgery are summarized in Table 3. Two patients who received immunotherapy as the first-line treatment without surgery showed overall survival of 34 and 18 months, respectively. One of them had distant LN and adrenal gland metastases at presentation and received Nivolumab for 24 months (3 mg/kg, biweekly) until disease progression (TPS for PD-L1 was 0%). This patient is still currently alive and undergoing clinical trial. No immunotherapy related adverse effects were reported in either patients. Two patients who received conventional chemotherapy and/or radiotherapy as the first-line treatment survived 10 and 5 months, respectively. One patient who received supportive care only due to old age died 6 months after diagnosis.

**Table 3:** Outcomes of patients not undergoing surgery for primary malignant melanoma of esophagus

No	Age	Sex	Tumor morphology	Metastases	BRAF	PD-L1 (TPS)	1st treatment	Time to progression	2nd treatment	Time to progression	3rd treatment	Time to progression	Overall survival (months)	Outcome
1	64	M	Diffusely infiltrative	LN, adrenal gland	N/A	0%	Nivolumab	24 months	DPT	2 months	Trastuzumab + Deruxtecan (Clinical trial)	Alive by 2021.06.30	34	Alive
2	82	M	Mass-forming	None	N/A	N/A	Pembrolizumab	2 months	DPT	8 months	Supportive care		18	Dead
3	63	M	Mass-forming	Lung, liver, LN, adrenal gland, thyroid	N/A	N/A	DBPT	2 months	IFN α	2 months	Supportive care		10	Dead
4	83	M	Mass-forming	None	N/A	N/A	Supportive care						6	Dead
5	60	M	Mass-forming	Bone, LN	Not detected	N/A	RT	1 week	DPT	2 months	Ipilimumab	1 month	5	Dead

*PD-L1* programmed death-ligand 1, *TPS* tumor proportion score, *M* male, *LN* lymph node, *DPT* Dacarbazine + Cisplatin ± Tamoxifen, *DBPT* Dacarbazine + Carmustine + Cisplatin ± Tamoxifen, *RT* radiotherapy, *IFN-α* interferon alpha, *N/A* not available

**Fig. 2:**
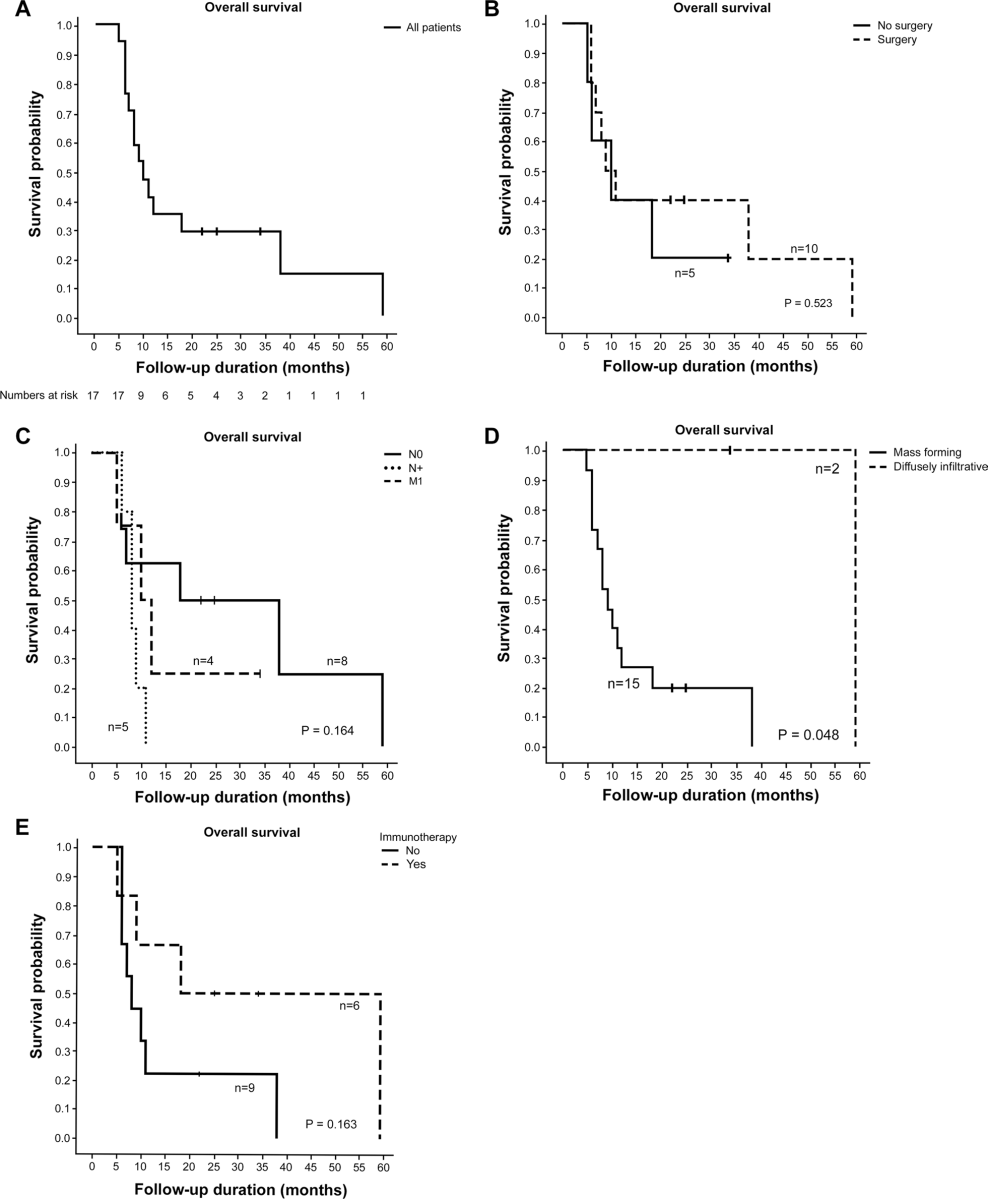
Kaplan–Meier overall survival curves in all patients (**A**) and according to treatment with or without surgery (**B**), stage groupings (**C**), gross tumor morphology (**D**), and treatment with or without immunotherapy (**E**)

The Kaplan–Meier curve for overall survival in all 17 patients is shown in Fig. 2A. The median follow-up time was 34.0 months (95% confidence interval (CI): 14.7 – 53.3 months). The median survival was 10 months (95% CI: 6.0 – 14.0 months) and the estimated probability of one-year and three-year survival was 35.3% and 29.4%, respectively. There was no statistical difference in overall survival between those who received surgery and those who did not (Fig. 2B). There was no statistically significant difference in overall survival between patients with localized, node positive, and metastatic disease (Fig. 2C). Patients with diffusely infiltrative tumor morphology showed significantly better overall survival compared to patients with mass-forming tumor morphology (Fig. 2D, *P* = 0.048). There was no statistically significant difference in overall survival between patients who received immunotherapy at any point (adjuvant or palliative) during their treatment course (patient number 1, 3, 6 in Table 2 and number 1, 2, 5 in Table 3) and those who did not (Fig. 2E). There was no mutation identified in exon 15 among the six patients who underwent PCR sequencing for BRAF.

## Discussion

Because PMME is a rare disease entity, its clinical features and treatment outcomes have not been fully defined. In the present single-center retrospective cohort study, we analyzed the clinical characteristics and survival outcomes of 17 PMME patients. We found that the majority of PMME patients are male (15/17, 88.2%), mainly complain of dysphagia (9/17, 52.9%) and present with large dark pigmented mass (13/17, 76.5%) at lower esophagus (11/17, 64.7%). Although surgery was performed in 58.8% of cases (n = 10), no significant improvement of overall survival was found compared to those who underwent non-surgical treatments (n = 5) (*P* = 0.523). Having a diffusely infiltrative tumor morphology (n = 2) was significantly associated with better overall survival than mass-forming tumor morphology (n = 15) (*P* = 0.048). Two patients who received immunotherapy as the first-line treatment without surgery showed overall survival of 34 and 18 months, respectively.

PMME is notorious for its aggressive behavior. Sabanathan et al. [1] reported five-year survival of 4.2% after radical surgical resection in the review of 139 cases reported worldwide. In a recent multicenter study from China with 70 PMME patients undergoing surgery, the median overall survival was 13.5 months and the median disease-free survival was 5.9 months [15]. In a previous study by Ahn et al. [2] which analyzed 19 South Korean PMME patients, the median overall survival was 12 months. In the present study, the estimated overall survival was 10 months (95% CI: 6.0 – 14.0 months). Previous studies have shown conflicting results on the effect of surgery on survival outcome. While some studies have advocated surgery as a treatment of choice for either pallation or cure [1, 4–7, 16], relatively large-scale studies by Weiner et al. [3] (n = 56) and Cheung et al. [17] (n = 39) failed to show significant association between surgery and prolonged overall survival. In the present study, whether or not the patient underwent surgery was not associated with overall survival (Fig. 2B, *P* = 0.523). We assume that this is mainly due to the extremely aggressive biology of PMME. Even in clinically localized diseases, early systemic dissemination at microscopic level could occur in PMME patients. In fact, all surgically treated patients experienced recurrence in our study. Consistently, there was no survival difference between patients with clinically localized disease (n = 8) and patients with node positive (n = 5) or metastatic diseases (n = 4) (Fig. 2C, *P* = 0.164). Furthermore, esophagectomy is known for its high risk of post-operative morbidities [18] and diminished quality of life after surgery [19]. Given the aggressive behavior of PMME and equivocal efficacy of surgery as well as the aforementioned post-operative morbidity and quality of life issues, further large-scale studies are required to determine the value of surgery as the first-line treatment modality for patients with PMME. To avoid possible bias and overcome the limitation of this study, it would be desirable if multivariate analysis can be performed in future studies with the adjustments for patients’ age, performance status and adjuvant treatment.

Ahn et al. [2] previously reported that regarding gross tumor morphology, patients with flat pigmented pattern tumor showed significantly better overall survival compared to those with mass-forming pattern. Consistent results were found in our study (Fig. 2D, *P* = 0.048). However, these results should be interpretated with caution because in both studies, the number of patients with infiltrative morphology was very small. Interestingly, in a patient with diffusely infiltrative tumor morphology who underwent surgery (patient number 1 in Table 2), pathologic tumor size was only 0.8 cm and the rest of the pigmented infiltration was benign melanosis. Given that PMME usually presents with large mass, it is possible that the favorable outcomes of diffusely infiltrative type tumors could have been due to small tumor volume.

The diagnosis of PMME can be especially challenging when the tumor is amelanotic. Amelanotic PMME can be pathologically suggested when there is no melanin granule inside the tumor cells but when IHC staining is positive for human melanin black 45 or S-100 and negative for cytokeratin [20]. The prevalence of amelanotic variant of PMME is estimated to be 10–25% [21]. In the present study, four cases (23.5%) were amelanotic subtype. Clinicians should be aware that not all melanomas are dark pigmented and pathologic diagnosis may change from poorly differentiated carcinoma to malignant melanoma after IHC investigations. The prognostic value of amelanotic gross appearance is unclear. In this study, there was no significance difference of overall survival between melanotic and amelanotic subtypes.

Immunotherapy has been greatly successful in the treatment of cutaneous melanoma [22]. However, previous studies have reported lower response rates of immunotherapy for mucosal melanoma compared to those for cutaneous melanoma. In a pooled analysis of clinical trials by D’Angelo et al. [23], the median progression-free survival among patients who received Nivolumab monotherapy was 3.0 months and 6.2 months for mucosal and cutaneous melanoma, respectively. A combination of Nivolumab and Ipilimumab showed better outcomes with the median progression-free survival of 5.9 months and 11.7 months for mucosal and cutaneous melanoma, respectively. In the present study, we identified two patients who received adjuvant Pembrolizumab after surgery. Although statistical analysis was not feasible due to small number of cases, disease-free survival in patients who received adjuvant Pembrolizumab after surgery did not exceed the median disease-free survival of surgically treated patients not undergoing adjuvant immunotherapy (4 months). Notably, one patient with distant LN and adrenal gland metastases received 24 months of Nivolumab as first-line therapy and succeeded in long-term survival of 34 months (Table 3). As other recent case studies consistently report the effectiveness of immunotherapy for metastatic PMME [9, 11], further large scale studies are warranted to confirm the validity of immunotherapy for PMME. To date, it is unclear whether PD-L1 expression can be a predictive marker for immunotherapy response for mucosal melanoma [23, 24]. In the present study, patient with 30% of PD-L1 expression showed comparable disease-free survival to patient with 1% of PD-L1 expression after adjuvant Pembrolizumab. In addition, the patient who remained progression-free for 24 months on Nivolumab monotherapy had 0% PD-L1 expression. Further studies are needed to clarify the potential role of PD-L1 as a predictive marker for immunotherapy response in patients with PMME. While BRAF mutation occurs up to 50% in cutaneous melanoma [25], its incidence has been reported to be 4–12% in mucosal melanoma [26–28]. This difference may be attributed to the absence of ultraviolet light exposure for carcinogenesis in mucosal melanoma. In the present study, six patients with PMME were tested for BRAF mutation, which was not found in any one of them.

Mucosal melanoma is generally considered to be chemotherapy-resistant [24, 29]. However, PMME patients may benefit from novel therapeutic options such as combination of immunotherapy with conventional chemotherapy [30]. In the present study, V777L HER2 mutation was identified in patient number 1 in Table 2 through next generation sequencing study. Following Nivolumab and conventional chemotherapy, the patient received Trastuzumab-Deruxtecan, which is a monoclonal antibody-topoisomerase inhibitor conjugate, and showed at least 8 months of progression-free survival. Further studies are needed to diversify the treatment options for PMME patients.

There are evident limitations to this study. This was a retrospective study performed at a single tertiary referral center. As the number of cases was small, comprehensive comparative analyses were limited and conclusive statements could not be made.

## Conclusions

PMME is a lethal disease with distinct clinical characteristics. As the treatment for PMME is not standardized and the efficacy of surgery is still controversial, further large-scale studies are required regarding novel treatment strategies such as immunotherapy for patients with PMME.

## Data Availability

The datasets used and/or analysed during the current study are available from the corresponding author on reasonable request.

## Abbreviations

PMME: Primary malignant melanoma of esophagus
PD: Programmed death
IRB: Institutional review board
IT: Incisor teeth
LNM: Lymph node metastases
PCR: Polymerase chain reaction
IHC: Immunohistochemical
PD-L1: Programmed death-ligand 1
TPS: Tumor proportion score
IFN-α: Interferon-alpha
CI: Confidence interval
